# Dichlorido(4-meth­oxy-2-{[2-(piperazin-4-ium-1-yl)eth­yl]imino­meth­yl}phenol­ate)cadmium

**DOI:** 10.1107/S1600536811022100

**Published:** 2011-06-18

**Authors:** Muhammad Saleh Salga, Hamid Khaledi, Hapipah Mohd Ali

**Affiliations:** a Department of Chemistry, University of Malaya, 50603 Kuala Lumpur, Malaysia

## Abstract

In the title compound, [CdCl_2_(C_14_H_21_N_3_O_2_)], the Schiff base ligand chelates the Cd^II^ ion in an *N,N,O*-tridentate fashion. Two Cl atoms complete a distorted square-pyramidal coordination environment around the metal atom. In the crystal, adjacent mol­ecules are linked through C—H⋯π inter­actions into infinite chains along the *a* axis. The mol­ecules are further connected into a three-dimensional network *via* N—H⋯O, N—H⋯Cl and C—H⋯Cl inter­actions. The ethyl­ene group is disordered over two sets of sites in a 0.520 (10):0.480 (10) ratio.

## Related literature

For similar structures, see: Mukhopadhyay *et al.* (2003 ▶); Xu *et al.* (2008 ▶); Saleh Salga *et al.* (2010 ▶). For a description of the geometry of complexes with five-coordinated metal ions, see: Addison *et al.* (1984 ▶).
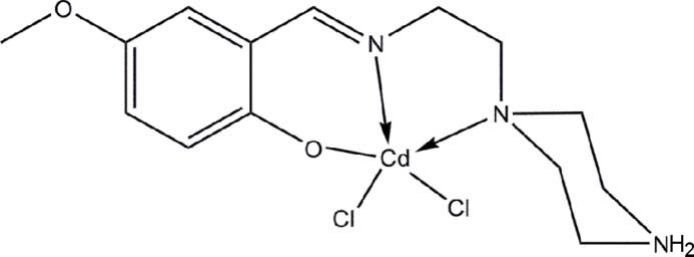


## Experimental

### 

#### Crystal data

[CdCl_2_(C_14_H_21_N_3_O_2_)]
*M* *_r_* = 446.64
Monoclinic, 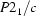

*a* = 10.1173 (9) Å
*b* = 16.2686 (15) Å
*c* = 10.3486 (10) Å
β = 103.069 (1)°
*V* = 1659.2 (3) Å^3^
*Z* = 4
Mo *K*α radiation
μ = 1.65 mm^−1^
*T* = 100 K
0.25 × 0.18 × 0.04 mm

#### Data collection

Bruker APEXII CCD diffractometer
Absorption correction: multi-scan (*SADABS*; Sheldrick, 1996 ▶) *T*_min_ = 0.684, *T*_max_ = 0.937
14540 measured reflections
3624 independent reflections
3138 reflections with *I* > 2σ(*I*)
*R*_int_ = 0.040

#### Refinement

*R*[*F* ^2^ > 2σ(*F* ^2^)] = 0.031
*wR*(*F* ^2^) = 0.061
*S* = 1.07
3624 reflections
225 parameters
5 restraints
H atoms treated by a mixture of independent and constrained refinement
Δρ_max_ = 0.65 e Å^−3^
Δρ_min_ = −0.78 e Å^−3^

### 

Data collection: *APEX2* (Bruker, 2007 ▶); cell refinement: *SAINT* (Bruker, 2007 ▶); data reduction: *SAINT*; program(s) used to solve structure: *SHELXS97* (Sheldrick, 2008 ▶); program(s) used to refine structure: *SHELXL97* (Sheldrick, 2008 ▶); molecular graphics: *X-SEED* (Barbour, 2001 ▶); software used to prepare material for publication: *SHELXL97* and *publCIF* (Westrip, 2010 ▶).

## Supplementary Material

Crystal structure: contains datablock(s) I, global. DOI: 10.1107/S1600536811022100/xu5230sup1.cif

Structure factors: contains datablock(s) I. DOI: 10.1107/S1600536811022100/xu5230Isup2.hkl

Additional supplementary materials:  crystallographic information; 3D view; checkCIF report

## Figures and Tables

**Table 1:** Selected bond lengths (Å)

Cd1—O1	2.241 (2)
Cd1—N1	2.245 (3)
Cd1—N2	2.475 (2)
Cd1—Cl1	2.4584 (8)
Cd1—Cl2	2.4797 (8)

**Table 2:** Hydrogen-bond geometry (Å, °) *Cg*1 is the centroid of the C1–C6 ring.

*D*—H⋯*A*	*D*—H	H⋯*A*	*D*⋯*A*	*D*—H⋯*A*
N3—H3*A*⋯O1^i^	0.92 (2)	1.80 (2)	2.705 (3)	166 (4)
N3—H3*B*⋯Cl2^ii^	0.90 (2)	2.33 (2)	3.222 (3)	174 (4)
C9—H9*A*⋯Cl1^iii^	0.99	2.64	3.454 (8)	139
C8—H8*A*⋯Cl2^iv^	0.98	2.82	3.777 (3)	167
C8—H8*B*⋯Cl1^v^	0.98	2.76	3.514 (3)	134
C13—H13*A*⋯Cl1^i^	0.99	2.71	3.542 (4)	142
C12—H12*A*⋯*Cg*1^vi^	0.99	2.48	3.408 (4)	156
C9′—H9*D*⋯*Cg*1^iv^	0.99	2.72	3.620 (8)	151

Symmetry codes: (i) 
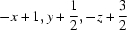
; (ii) 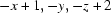; (iii) 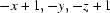; (iv) 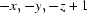; (v) 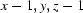; (vi) 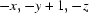.

**Fig. 1.**
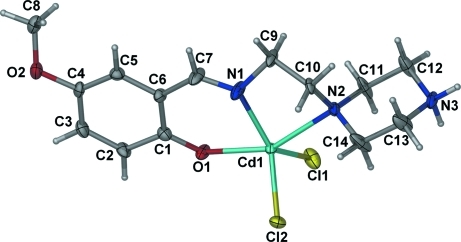
Thermal ellipsoid plot of the title compound at the 50% probability level. Hydrogen atoms are drawn as spheres of arbitrary radius. Only the major disordered component is shown.

## supplementary crystallographic information

### Comment

The ligands containing a piperazine ring have been reported to possess ambidentate character, capable of binding metal centers through one or both piperazine N atoms (Mukhopadhyay *et al.*, 2003; Xu *et al.*, 2008; Saleh Salga *et al.*, 2010). The coordination mode depends largely on the conformation (chair or boat) adopted by the ring which, in turn, depends on the metal salt entity and remote substituations in the ligand. In the present structure, the piperazine ring assumes a chair conformation and employs only one of the ring N atoms to bind to the metal center. The Schiff base ligand chelates the metal atom in an *N,N,O*-mode, along with two Cl atoms makes a distorted square-pyramidal cadmium(II) complex. The distortion from the ideal geometry is evident from the τ index of 0.30 (τ is 0 for an ideal square-pyramid and is 1 for a perfect trigonal-bipyramid, Addison *et al.*, 1984). The other ring N atom stays away from the chelation, and is protonated to keep the electronutrality of the molecule, thus the complex can be described as a zwitterion. In the crystal, C—H···π interactions link the molecules into infinite chains along the *a* axis and these are connected into a three-dimensional network *via* N—H···O, N—H···Cl and C—H···Cl hydrogen bonds (Table 1).

### Experimental

A mixture of 5-methoxysalicylaldehyde (0.35 g, 2.3 mmol) and aminoethylpiperazine (0.3 g, 2.3 mmol) in ethanol (20 ml) was refluxed for 2 h, followed by addition of a solution of cadmium(II) chloride (0.42 g, 2.3 mmol) in a minimum amount of ethanol. The resulting solution was refluxed for 1 h and then left at room temperature for one day to give the X-ray quality crystals of the title compound.

### Refinement

The C-bound hydrogen atoms were placed at calculated positions and refined as riding atoms with C—H distances of 0.95 (aryl), 0.98 (methyl) and 0.99 Å (methylene). The N-bound hydrogen atoms were located in a difference Fourier map and refined with distance restraint of N—H 0.91 (2) Å. For all hydrogen atoms *U*iso(H) were set to 1.2 (1.5 for methyl)*U*eq(carrier atom). C9 and C10 were found to be disordered with two positions being resolved for each of the atoms. From anisotropic refinement, the site occupancy factor of the major component refined is 0.52 (1). The corresponding bond distances involving the disordered atoms were restrained to be equal with the SADI command in *SHELXL97* (Sheldrick, 2008).

### Crystal data

**Table:**

[CdCl_2_(C_14_H_21_N_3_O_2_)]	*F*(000) = 896
*M**_r_* = 446.64	*D*_x_ = 1.788 Mg m^−^^3^
Monoclinic, *P*2_1_/*c*	Mo *K*α radiation, λ = 0.71073 Å
Hall symbol: -P 2ybc	Cell parameters from 4675 reflections
*a* = 10.1173 (9) Å	θ = 2.4–30.3°
*b* = 16.2686 (15) Å	µ = 1.65 mm^−^^1^
*c* = 10.3486 (10) Å	*T* = 100 K
β = 103.069 (1)°	Plate, yellow
*V* = 1659.2 (3) Å^3^	0.25 × 0.18 × 0.04 mm
*Z* = 4	

### Data collection

**Table:**

Bruker APEXII CCD diffractometer	3624 independent reflections
Radiation source: fine-focus sealed tube	3138 reflections with *I* > 2σ(*I*)
graphite	*R*_int_ = 0.040
φ and ω scans	θ_max_ = 27.0°, θ_min_ = 2.1°
Absorption correction: multi-scan (*SADABS*; Sheldrick, 1996)	*h* = −12→12
*T*_min_ = 0.684, *T*_max_ = 0.937	*k* = −20→20
14540 measured reflections	*l* = −12→13

### Refinement

**Table:**

Refinement on *F*^2^	Primary atom site location: structure-invariant direct methods
Least-squares matrix: full	Secondary atom site location: difference Fourier map
*R*[*F*^2^ > 2σ(*F*^2^)] = 0.031	Hydrogen site location: inferred from neighbouring sites
*wR*(*F*^2^) = 0.061	H atoms treated by a mixture of independent and constrained refinement
*S* = 1.07	*w* = 1/[σ^2^(*F*_o_^2^) + (0.0115*P*)^2^ + 3.0473*P*] where *P* = (*F*_o_^2^ + 2*F*_c_^2^)/3
3624 reflections	(Δ/σ)_max_ < 0.001
225 parameters	Δρ_max_ = 0.65 e Å^−^^3^
5 restraints	Δρ_min_ = −0.78 e Å^−^^3^

### Special details

**Table:**

Geometry. All e.s.d.'s (except the e.s.d. in the dihedral angle between two l.s. planes) are estimated using the full covariance matrix. The cell e.s.d.'s are taken into account individually in the estimation of e.s.d.'s in distances, angles and torsion angles; correlations between e.s.d.'s in cell parameters are only used when they are defined by crystal symmetry. An approximate (isotropic) treatment of cell e.s.d.'s is used for estimating e.s.d.'s involving l.s. planes.
Refinement. Refinement of *F*^2^ against ALL reflections. The weighted *R*-factor *wR* and goodness of fit *S* are based on *F*^2^, conventional *R*-factors *R* are based on *F*, with *F* set to zero for negative *F*^2^. The threshold expression of *F*^2^ > σ(*F*^2^) is used only for calculating *R*-factors(gt) *etc*. and is not relevant to the choice of reflections for refinement. *R*-factors based on *F*^2^ are statistically about twice as large as those based on *F*, and *R*- factors based on ALL data will be even larger.

### Fractional atomic coordinates and isotropic or equivalent isotropic displacement parameters (Å^2^)

**Table:**

	*x*	*y*	*z*	*U*_iso_*/*U*_eq_	Occ. (<1)
Cd1	0.34107 (2)	−0.052048 (13)	0.68131 (2)	0.01663 (7)	
Cl1	0.56656 (8)	−0.11579 (5)	0.74780 (10)	0.0354 (2)	
Cl2	0.23070 (8)	−0.07175 (5)	0.87000 (8)	0.02395 (17)	
O1	0.2602 (2)	−0.15436 (13)	0.5407 (2)	0.0251 (5)	
O2	−0.1051 (2)	−0.17553 (14)	0.0579 (2)	0.0268 (5)	
N1	0.2318 (3)	0.02551 (17)	0.5110 (3)	0.0360 (8)	
N2	0.4238 (3)	0.08960 (15)	0.7371 (3)	0.0201 (6)	
N3	0.6073 (3)	0.20036 (17)	0.9144 (3)	0.0282 (7)	
H3A	0.644 (3)	0.2509 (14)	0.941 (4)	0.034*	
H3B	0.647 (3)	0.1620 (18)	0.972 (3)	0.034*	
C1	0.1647 (3)	−0.15272 (19)	0.4325 (3)	0.0213 (7)	
C2	0.1038 (3)	−0.22708 (19)	0.3799 (4)	0.0250 (7)	
H2	0.1240	−0.2756	0.4316	0.030*	
C3	0.0166 (3)	−0.2327 (2)	0.2574 (4)	0.0259 (7)	
H3	−0.0201	−0.2846	0.2257	0.031*	
C4	−0.0181 (3)	−0.16303 (19)	0.1797 (3)	0.0199 (6)	
C5	0.0301 (3)	−0.08803 (19)	0.2301 (3)	0.0221 (7)	
H5	0.0028	−0.0398	0.1792	0.027*	
C6	0.1199 (3)	−0.08094 (18)	0.3567 (3)	0.0198 (6)	
C7	0.1562 (4)	0.0024 (2)	0.3998 (4)	0.0336 (9)	
H7	0.1201	0.0448	0.3390	0.040*	
C8	−0.1108 (3)	−0.1114 (2)	−0.0365 (3)	0.0277 (7)	
H8A	−0.1539	−0.0629	−0.0077	0.042*	
H8B	−0.1639	−0.1297	−0.1230	0.042*	
H8C	−0.0186	−0.0974	−0.0440	0.042*	
C9	0.2794 (9)	0.1146 (3)	0.5179 (6)	0.027 (2)	0.520 (10)
H9A	0.3623	0.1195	0.4828	0.033*	0.520 (10)
H9B	0.2081	0.1500	0.4639	0.033*	0.520 (10)
C10	0.3083 (6)	0.1410 (3)	0.6602 (6)	0.0207 (17)	0.520 (10)
H10A	0.2267	0.1333	0.6965	0.025*	0.520 (10)
H10B	0.3334	0.1999	0.6674	0.025*	0.520 (10)
C9'	0.2199 (7)	0.1130 (4)	0.5540 (8)	0.0213 (18)	0.480 (10)
H9C	0.1786	0.1483	0.4775	0.026*	0.480 (10)
H9D	0.1646	0.1166	0.6215	0.026*	0.480 (10)
C10'	0.3645 (7)	0.1373 (4)	0.6114 (7)	0.0227 (19)	0.480 (10)
H10C	0.3686	0.1969	0.6314	0.027*	0.480 (10)
H10D	0.4195	0.1270	0.5452	0.027*	0.480 (10)
C11	0.5665 (4)	0.0977 (2)	0.7352 (4)	0.0374 (10)	
H11A	0.5772	0.0875	0.6438	0.045*	
H11B	0.6185	0.0549	0.7932	0.045*	
C12	0.6273 (4)	0.1815 (2)	0.7807 (4)	0.0363 (9)	
H12A	0.7255	0.1814	0.7821	0.044*	
H12B	0.5833	0.2244	0.7175	0.044*	
C13	0.4637 (4)	0.1933 (2)	0.9208 (4)	0.0407 (10)	
H13A	0.4098	0.2365	0.8652	0.049*	
H13B	0.4550	0.2012	1.0134	0.049*	
C14	0.4095 (4)	0.1085 (2)	0.8714 (4)	0.0441 (11)	
H14A	0.4583	0.0662	0.9329	0.053*	
H14B	0.3123	0.1054	0.8735	0.053*	

### Atomic displacement parameters (Å^2^)

**Table:**

	*U*^11^	*U*^22^	*U*^33^	*U*^12^	*U*^13^	*U*^23^
Cd1	0.01874 (11)	0.01363 (11)	0.01584 (12)	−0.00060 (9)	0.00039 (8)	0.00174 (9)
Cl1	0.0207 (4)	0.0231 (4)	0.0576 (6)	0.0037 (3)	−0.0014 (4)	0.0042 (4)
Cl2	0.0276 (4)	0.0202 (4)	0.0259 (4)	−0.0011 (3)	0.0097 (3)	0.0048 (3)
O1	0.0382 (13)	0.0120 (10)	0.0204 (12)	0.0043 (9)	−0.0033 (10)	0.0012 (9)
O2	0.0250 (12)	0.0280 (13)	0.0245 (13)	−0.0034 (10)	−0.0007 (10)	−0.0050 (10)
N1	0.060 (2)	0.0154 (14)	0.0215 (16)	0.0037 (14)	−0.0132 (15)	−0.0005 (12)
N2	0.0188 (13)	0.0163 (12)	0.0217 (15)	−0.0021 (10)	−0.0028 (11)	0.0021 (11)
N3	0.0386 (17)	0.0143 (13)	0.0228 (17)	−0.0021 (12)	−0.0119 (13)	−0.0002 (11)
C1	0.0189 (15)	0.0175 (15)	0.0274 (18)	0.0022 (12)	0.0050 (13)	0.0013 (13)
C2	0.0249 (17)	0.0140 (15)	0.033 (2)	0.0000 (13)	0.0008 (14)	0.0041 (14)
C3	0.0233 (16)	0.0178 (15)	0.035 (2)	−0.0022 (13)	0.0036 (15)	−0.0054 (14)
C4	0.0135 (14)	0.0259 (16)	0.0187 (16)	0.0003 (12)	0.0004 (12)	−0.0044 (13)
C5	0.0251 (16)	0.0184 (15)	0.0226 (18)	0.0016 (13)	0.0049 (13)	0.0031 (13)
C6	0.0256 (16)	0.0150 (14)	0.0179 (17)	0.0009 (12)	0.0032 (13)	−0.0023 (12)
C7	0.053 (2)	0.0176 (16)	0.0223 (19)	0.0073 (16)	−0.0088 (17)	0.0040 (14)
C8	0.0235 (17)	0.0355 (19)	0.0224 (19)	0.0033 (14)	0.0015 (14)	−0.0050 (15)
C9	0.040 (5)	0.012 (3)	0.021 (4)	0.003 (3)	−0.010 (3)	−0.004 (3)
C10	0.020 (3)	0.012 (3)	0.027 (4)	−0.003 (2)	−0.002 (3)	−0.004 (3)
C9'	0.023 (4)	0.020 (4)	0.020 (4)	0.007 (3)	0.002 (3)	0.000 (3)
C10'	0.031 (4)	0.014 (3)	0.021 (4)	−0.005 (3)	0.004 (3)	0.001 (3)
C11	0.034 (2)	0.034 (2)	0.053 (3)	−0.0217 (16)	0.0285 (19)	−0.0279 (18)
C12	0.0297 (19)	0.0302 (19)	0.057 (3)	−0.0137 (15)	0.0255 (18)	−0.0224 (18)
C13	0.060 (3)	0.0272 (19)	0.046 (3)	−0.0171 (18)	0.035 (2)	−0.0180 (17)
C14	0.062 (3)	0.0273 (19)	0.058 (3)	−0.0221 (18)	0.044 (2)	−0.0194 (19)

### Geometric parameters (Å, °)

**Table:**

Cd1—O1	2.241 (2)	C5—H5	0.9500
Cd1—N1	2.245 (3)	C6—C7	1.448 (4)
Cd1—N2	2.475 (2)	C7—H7	0.9500
Cd1—Cl1	2.4584 (8)	C8—H8A	0.9800
Cd1—Cl2	2.4797 (8)	C8—H8B	0.9800
O1—C1	1.303 (4)	C8—H8C	0.9800
O2—C4	1.380 (4)	C9—C10	1.498 (7)
O2—C8	1.421 (4)	C9—H9A	0.9900
N1—C7	1.285 (4)	C9—H9B	0.9900
N1—C9'	1.505 (6)	C10—H10A	0.9900
N1—C9	1.524 (6)	C10—H10B	0.9900
N2—C11	1.455 (4)	C9'—C10'	1.503 (7)
N2—C14	1.462 (4)	C9'—H9C	0.9900
N2—C10	1.509 (6)	C9'—H9D	0.9900
N2—C10'	1.518 (6)	C10'—H10C	0.9900
N3—C13	1.474 (5)	C10'—H10D	0.9900
N3—C12	1.476 (5)	C11—C12	1.525 (4)
N3—H3A	0.918 (18)	C11—H11A	0.9900
N3—H3B	0.897 (18)	C11—H11B	0.9900
C1—C2	1.410 (4)	C12—H12A	0.9900
C1—C6	1.423 (4)	C12—H12B	0.9900
C2—C3	1.373 (5)	C13—C14	1.529 (5)
C2—H2	0.9500	C13—H13A	0.9900
C3—C4	1.388 (5)	C13—H13B	0.9900
C3—H3	0.9500	C14—H14A	0.9900
C4—C5	1.372 (4)	C14—H14B	0.9900
C5—C6	1.420 (4)		
			
O1—Cd1—N1	82.23 (9)	O2—C8—H8A	109.5
O1—Cd1—Cl1	92.75 (6)	O2—C8—H8B	109.5
N1—Cd1—Cl1	135.90 (10)	H8A—C8—H8B	109.5
O1—Cd1—N2	153.88 (8)	O2—C8—H8C	109.5
N1—Cd1—N2	74.96 (9)	H8A—C8—H8C	109.5
Cl1—Cd1—N2	94.82 (6)	H8B—C8—H8C	109.5
O1—Cd1—Cl2	104.60 (6)	C10—C9—N1	107.9 (5)
N1—Cd1—Cl2	117.46 (9)	C10—C9—H9A	110.1
Cl1—Cd1—Cl2	106.24 (3)	N1—C9—H9A	110.1
N2—Cd1—Cl2	97.20 (7)	C10—C9—H9B	110.1
C1—O1—Cd1	129.33 (19)	N1—C9—H9B	110.1
C4—O2—C8	116.0 (2)	H9A—C9—H9B	108.4
C7—N1—C9'	118.1 (4)	C9—C10—N2	108.4 (5)
C7—N1—C9	116.0 (4)	C9—C10—H10A	110.0
C9'—N1—C9	29.7 (3)	N2—C10—H10A	110.0
C7—N1—Cd1	128.7 (2)	C9—C10—H10B	110.0
C9'—N1—Cd1	111.0 (3)	N2—C10—H10B	110.0
C9—N1—Cd1	113.8 (3)	H10A—C10—H10B	108.4
C11—N2—C14	107.8 (3)	C10'—C9'—N1	103.3 (5)
C11—N2—C10	126.5 (4)	C10'—C9'—H9C	111.1
C14—N2—C10	98.8 (4)	N1—C9'—H9C	111.1
C11—N2—C10'	98.2 (4)	C10'—C9'—H9D	111.1
C14—N2—C10'	127.0 (4)	N1—C9'—H9D	111.1
C10—N2—C10'	32.4 (3)	H9C—C9'—H9D	109.1
C11—N2—Cd1	111.46 (19)	C9'—C10'—N2	111.4 (6)
C14—N2—Cd1	108.48 (19)	C9'—C10'—H10C	109.3
C10—N2—Cd1	102.3 (3)	N2—C10'—H10C	109.3
C10'—N2—Cd1	103.2 (3)	C9'—C10'—H10D	109.3
C13—N3—C12	111.8 (3)	N2—C10'—H10D	109.3
C13—N3—H3A	113 (2)	H10C—C10'—H10D	108.0
C12—N3—H3A	110 (2)	N2—C11—C12	114.2 (3)
C13—N3—H3B	103 (2)	N2—C11—H11A	108.7
C12—N3—H3B	110 (2)	C12—C11—H11A	108.7
H3A—N3—H3B	109 (3)	N2—C11—H11B	108.7
O1—C1—C2	119.3 (3)	C12—C11—H11B	108.7
O1—C1—C6	124.9 (3)	H11A—C11—H11B	107.6
C2—C1—C6	115.7 (3)	N3—C12—C11	110.1 (3)
C3—C2—C1	123.2 (3)	N3—C12—H12A	109.6
C3—C2—H2	118.4	C11—C12—H12A	109.6
C1—C2—H2	118.4	N3—C12—H12B	109.6
C2—C3—C4	120.4 (3)	C11—C12—H12B	109.6
C2—C3—H3	119.8	H12A—C12—H12B	108.2
C4—C3—H3	119.8	N3—C13—C14	109.8 (3)
C5—C4—O2	125.2 (3)	N3—C13—H13A	109.7
C5—C4—C3	119.0 (3)	C14—C13—H13A	109.7
O2—C4—C3	115.8 (3)	N3—C13—H13B	109.7
C4—C5—C6	121.4 (3)	C14—C13—H13B	109.7
C4—C5—H5	119.3	H13A—C13—H13B	108.2
C6—C5—H5	119.3	N2—C14—C13	113.8 (3)
C5—C6—C1	120.0 (3)	N2—C14—H14A	108.8
C5—C6—C7	115.2 (3)	C13—C14—H14A	108.8
C1—C6—C7	124.8 (3)	N2—C14—H14B	108.8
N1—C7—C6	127.5 (3)	C13—C14—H14B	108.8
N1—C7—H7	116.3	H14A—C14—H14B	107.7
C6—C7—H7	116.3		

### Hydrogen-bond geometry (Å, °)

**Table:**

Cg1 is the centroid of the C1–C6 ring.

**Table:**

*D*—H···*A*	*D*—H	H···*A*	*D*···*A*	*D*—H···*A*
N3—H3A···O1^i^	0.92 (2)	1.80 (2)	2.705 (3)	166 (4)
N3—H3B···Cl2^ii^	0.90 (2)	2.33 (2)	3.222 (3)	174 (4)
C9—H9A···Cl1^iii^	0.99	2.64	3.454 (8)	139
C8—H8A···Cl2^iv^	0.98	2.82	3.777 (3)	167
C8—H8B···Cl1^v^	0.98	2.76	3.514 (3)	134
C13—H13A···Cl1^i^	0.99	2.71	3.542 (4)	142
C12—H12A···Cg1^vi^	0.99	2.48	3.408 (4)	156
C9'—H9D···Cg1^iv^	0.99	2.72	3.620 (8)	151

Symmetry codes: (i) −*x*+1, *y*+1/2, −*z*+3/2; (ii) −*x*+1, −*y*, −*z*+2; (iii) −*x*+1, −*y*, −*z*+1; (iv) −*x*, −*y*, −*z*+1; (v) *x*−1, *y*, *z*−1; (vi) −*x*, −*y*+1, −*z*.
